# Development and Validation of a Prediction Model for Positive Findings of Preoperative Flexible Bronchoscopy in Patients with Peripheral Lung Cancer

**DOI:** 10.3390/curroncol30010025

**Published:** 2022-12-26

**Authors:** Dongyu Li, Zaishan Li, Shaolei Li, Hongbing Zhang, Siqing Yao, Yi Li, Jun Chen

**Affiliations:** 1 Department of Lung Cancer Surgery, Tianjin Medical University General Hospital, Tianjin 300052, China; 2 Yuncheng Central Hospital, Yuncheng 044000, China; 3 Department of Thoracic Surgery II, Key Laboratory of Carcinogenesis and Translational Research (Ministry of Education), Peking University Cancer Hospital and Institute, Beijing 100142, China

**Keywords:** flexible bronchoscopy, peripheral lung cancer, preoperative workup, preoperative evaluation, prediction model

## Abstract

(1) Background: It has yet to be determined whether preoperative flexible bronchoscopy (FB) should be routinely performed in patients with peripheral lung cancer. The aim of this study was to construct a model to predict the probability of positive FB findings, which would help assess the necessity of preoperative FB. (2) Methods: A total of 380 consecutive patients with peripheral lung cancer who underwent preoperative FB were recruited for this study. A prediction model was developed through univariate and multivariate logistic regression, with predictors including gender, age, body mass index (BMI), smoking, history of chronic lung diseases, respiratory symptoms, lesion size, lesion type, lesion location in the bronchi, and lesion location in the lobe. The predictive performance of the model was evaluated by validation using 1000 iterations of bootstrap resampling. Model discrimination was assessed using the area under the receiver operating characteristics curve (AUC), and calibration was assessed using the Brier score and calibration plots. (3) Results: The model suggested that male patients with respiratory symptoms, decreased BMI, solid lesions, and lesions located in lower-order bronchi were more likely to have positive FB findings. The AUC and Brier score of the model for internal validation were 0.784 and 0.162, respectively. The calibration curve for the probability of positive FB findings showed convincing concordance between the predicted and actual results. (4) Conclusions: Our prediction model estimated the pretest probability of positive FB findings in patients with peripheral lung cancers. Males and patients with lower BMI, the presence of respiratory symptoms, larger lesions, solid lesions, and lesions located in lower-order bronchi were associated with increased positive FB findings. The use of our model can be of assistance when making clinical decisions about preoperative FB.

## 1. Introduction

Lung cancer is the most prevalent cancer and the leading cause of cancer death worldwide, accounting for 11.4% of newly diagnosed cancer cases and 18.0% of all cancer-related deaths in 2020 [1]. Lung cancer may show morphological changes, such as endobronchial mass, mucosal abnormalities, and bronchial stenosis [2]. Flexible bronchoscopy (FB) is an important exploratory modality for lung cancer because it allows visual inspection of the airways [3].

The purposes of preoperative FB include not only diagnosing lung cancer but also determining whether there are macroscopic or microscopic changes that may affect surgical planning and whether specific preoperative treatments are needed to reduce potential complications. For patients with positive preoperative FB findings, corresponding intervention measures should be taken [4,5,6].

Guidelines for the diagnosis and treatment of lung cancer contain different recommendations for FB. The National Comprehensive Cancer Network (NCCN) guidelines recommend that preoperative FB should be routinely performed in patients with lung cancer who are to be treated surgically [7]. The European Society for Medical Oncology guidelines recommend bronchoscopy as the standard exploratory modality for stage I–III central lung cancer [8]. The American College of Chest Physicians guidelines do not recommend routine preoperative FB for indeterminate small pulmonary nodules except for pulmonary nodules with the presence of an air bronchogram [9]. However, the interdisciplinary guidelines of the German Respiratory Society and the German Cancer Society indicate that it is unclear whether bronchoscopy should be performed before planning surgery owing to the lack of sufficient data on patients with peripheral lung cancer less than 2 cm in diameter [3].

There is considerable controversy regarding the routine performance of preoperative FB in specific patients [10,11,12,13,14,15,16]. Previous studies have not established a clear consensus regarding bronchoscopy in patients with peripheral lung cancer who are to be treated surgically. Therefore, to explore the necessity of preoperative FB, this study aimed to construct a model to predict the probability of positive FB findings by retrospectively analyzing the preoperative FB findings and clinical factors in patients with peripheral lung cancer.

## 2. Materials and Methods

### 2.1. Study Population

This study was conducted in accordance with the Declaration of Helsinki (as revised in 2013) and was approved by the Institutional Review Board of the hospital (number/ID of ethics approval: YXLL2022011-2). 

**Figure 1:**
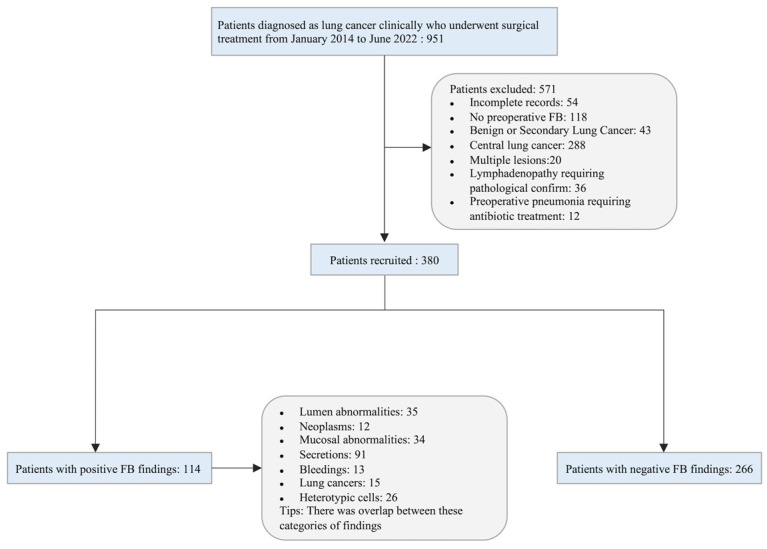
Flowchart of the study population.

A total of 951 consecutive patients diagnosed clinically with lung cancer who underwent surgical treatment at the Department of Thoracic Surgery of Yuncheng Central Hospital from January 2014 to June 2022 were reviewed. All patients underwent full oncological staging before surgery, excluding clinical stage IV disease. Patients who met the following criteria were recruited to the current study: (1) completed clinicopathology information; (2) performed preoperative FB; (3) pathologically diagnosed as primary lung cancer; (4) peripheral lung cancer; (5) single lesion; (6) no preoperative lymphadenopathy requiring pathological confirmation; (7) no preoperative pneumonia requiring treatment. Finally, 380 patients were included in the study. A flowchart of population selection in the study is shown in Figure 1. 

Demographic and clinical information of the study population was collected according to previous studies [3,9,10,15,17]. Patients were divided into FB findings-positive and FB findings-negative groups, and variables were compared between the groups. These variables included gender, age, body mass index (BMI), smoking, history of chronic lung diseases, respiratory symptoms, lesion size, lesion type, lesion location in the bronchi, and lesion location in the lobe. Patients were deemed to have a history of chronic lung disease if they had any of the following conditions: bronchial asthma, emphysema, or chronic obstructive pulmonary disease (COPD). Likewise, respiratory symptoms were identified when any of the following symptoms were present: cough, shortness of breath, or blood in the sputum. Lesion size was identified with the largest unidimensional size in the lung window (window width: 1500 HU, window level: −500 HU) of the chest computed tomography (CT) scan [18]. Lesion locations in the bronchi were classified with fifth-order bronchi (subsegmental bronchi) and sixth-order or higher bronchi (beyond the subsegmental bronchi). Central lung lesions were defined as tumors with locations limited to the trachea, bronchi, or segmental bronchi; peripheral lesions were defined as tumors with locations limited to the subsegmental or other distal bronchi and bronchioli [17,19,20].

### 2.2. Procedure of Preoperative Flexible Bronchoscopy

Preoperative FB was performed by a well-trained bronchoscopist with two nurses assisting in the supine position under local anesthesia with lidocaine. The equipment used for the FB was an Olympus BF-1T260 electronic bronchoscope (diameter 5.9 mm, clamp 2.8 mm). A complete examination of the larynx and tracheobronchial tree, including the subsegmental bronchi, was performed to detect any abnormal findings. Biopsies were taken using a brush, forceps, or cytologic washing. Biopsies were performed for patients with visualized intrabronchial lesions using forceps in most cases. A brush was used when their lesion was located at a site that was inaccessible to forceps. When no endobronchial lesion was visible through the bronchoscope, bronchial washing was performed in the corresponding segmental bronchus. Fluoroscopy was not used in this study. All FB findings were evaluated and recorded. In our study, no related complications, including hypoxemia, hemorrhage, or pneumothorax, were found.

### 2.3. Positive FB Findings

Positive FB findings, which were taken by the model and served as predicted outcomes, were defined as the detection of any morphological or histological abnormality in the airways, including (1) lumen abnormalities (obstruction or stenosis), neoplasms, mucosal abnormalities, secretions, or bleeding; (2) histological findings (lung cancers or heterotypic cells). By contrast, negative FB findings indicated that none of the above abnormalities were found.

### 2.4. Development and Validation of the Model

A model was developed to predict the probability of positive preoperative FB findings on the basis of the clinical characteristics of patients with peripheral lung cancers. The positive findings of preoperative FB were used as the outcome measures of the prediction model. Univariate logistic regression was used to select potential predictive variables of the model, and the variables with *p*-values less than 0.1 were retained as the candidates for multivariate logistic regression analysis. Finally, the method of “stepwise backward selection” was applied to identify variables left in the prediction model. The model was displayed in the form of a nomogram.

The model was subjected to 1000 iterations of bootstrap resampling for internal validation to assess predictive accuracy. The performance of the model was evaluated by discrimination and calibration; the area under the curve (AUC) in the receiver operating characteristic (ROC) analysis was used to assess the discriminative ability of the model, while the Brier score and calibration plots were used to evaluate the calibration ability of the model. In addition, decision curve analysis was used to determine the clinical usefulness of the model by quantifying the net benefits at different threshold probabilities.

### 2.5. Statistical Analysis

The baseline characteristics of the study population are described by means and standard deviations for continuous variables and absolute and relative frequencies for categorical variables. Student’s *t*-test was used to compare continuous variables, while the Chi-squared or Fisher exact test was used to compare differences in the percent of categorical variables, as appropriate. Two-sided *p*-values of <0.05 were considered statistically significant. All statistical analyses were performed using R version 4.1.1 (The R Foundation for Statistical Computing; http://www.R-project.org/ (accessed on 1 January 2022)).

## 3. Results

### 3.1. Baseline Characteristics of Study Population

There were 232 (61.1%) male and 148 (38.9%) female patients with an average age of 61.8 ± 8.6 years. There were 164 (43.2%) smokers and 216 (56.8%) non-smokers. A total of 84 patients (22.1%) had a history of chronic lung diseases, while 296 patients (77.9%) did not. The mean lesion size was 3.0 ± 1.9 cm, with 130 patients (34.2%) with a lesion size of >3.0 cm and 250 patients (65.8%) with a lesion size of ≤3.0 cm. There were 303 (79.7%) solid lesions and 77 (20.3%) subsolid lesions. The lesions were at fifth-order bronchi (level of subsegmental bronchi) in 234 (61.6%) cases and sixth-order or higher bronchi (beyond the level of subsegmental bronchi) in 146 (38.4%) cases. A total of 118 patients (31.1%) had lesions in the right upper lobe, 18 (4.7%) had lesions in the right middle lobe, 90 (23.7%) had lesions in the right lower lobe, 103 (27.1%) had lesions in the left upper lobe, and 51 (13.4%) had lesions in the left lower lobe.

### 3.2. Outcomes

Preoperative FB findings were positive in 114 patients (30.0%) and negative in 266 patients (70.0%). Positive preoperative FB findings included lumen abnormalities in 35 cases (30.7%), neoplasms in 12 cases (10.5%), mucosal abnormalities in 34 cases (29.8%), secretions in 91 cases (79.8%), bleeding in 13 cases (11.4%), and histological findings in 32 cases (28.1%). There was an overlap between these categories of positive findings.

There were significant differences (all *p*-values < 0.05) between the FB findings-positive group and the FB findings-negative group in terms of gender, age, BMI, smoking status, presence or absence of respiratory symptoms, lesion size, lesion type, and lesion location in the bronchi (Table 1). Compared with the FB findings-negative group, the FB findings-positive group had a greater proportion of males, older patients, smokers, and patients with decreased BMI, respiratory symptoms, larger lesions, solid lesions, or lesions located in lower-order bronchi.

### 3.3. Development and Validation of the Model

**Figure 2:**
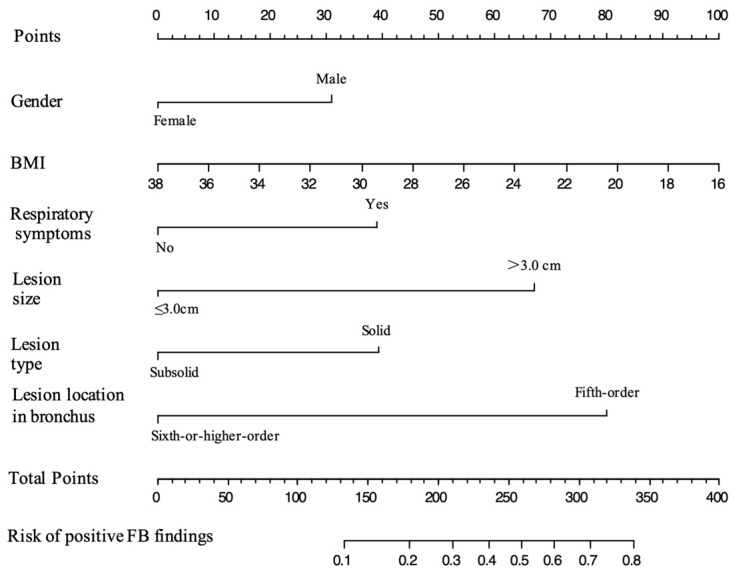
Nomogram for predicting the probability of positive FB findings in patients with peripheral lung cancer. The value of each predictive factor was given a score on the point scale axis. The total score was calculated by adding the scores for all the factors, and then, by projecting the total score to the lower total point scale, the probability of positive FB findings could be estimated.

According to the results of our analysis (Table 2 and Table 3), BMI (OR, 0.924; 95% CI, 0.854–0.996; *p*-value = 0.043), respiratory symptoms (*p*-value = 0.016), lesion size (OR, 3.212; 95% CI, 1.869–5.560; *p*-value < 0.001), and lesion location in the bronchi (OR, 0.248; 95% CI, 0.135–0.438; *p*-value < 0.001) were independent predictor factors for positive FB findings in patients with peripheral lung cancer. On the basis of the Akaike information criterion (AIC) achieved via stepwise backward selection, the gender and lesion type were also entered into the final model. Gender, BMI, respiratory symptoms, lesion size, lesion type, and lesion location in the bronchi were chosen to construct a model to predict the probability of positive FB findings in patients with peripheral lung cancer (Table 3). A nomogram was established according to the model (Figure 2).

**Figure 3:**
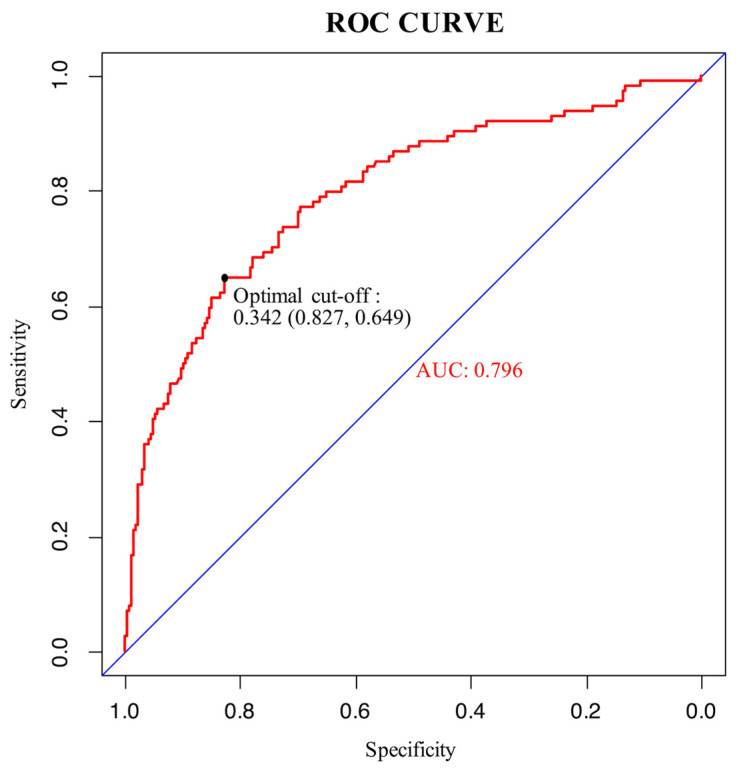
ROC curve of the model. The maximum Youden index of the ROC curve was 0.476, and the corresponding diagnostic cut-off value, sensitivity, and specificity of the model were 0.342, 64.9%, and 82.7%, respectively.

**Figure 4:**
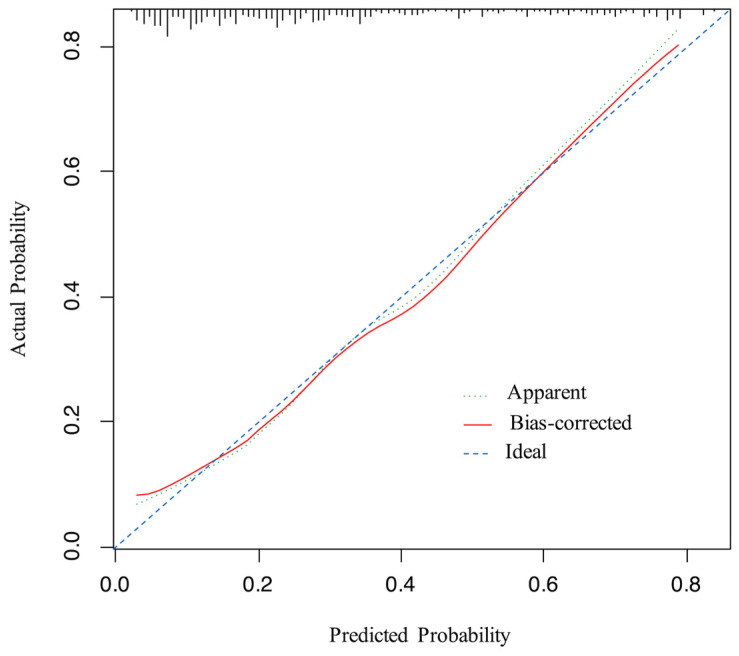
Calibration curve for the model. The x-axis represents the predicted probability of positive FB findings, the y-axis represents the actual probability of positive FB findings, and the ideal line is the diagonal of the graph, indicating that the predicted probability is utterly equal to the true likelihood, which is the ideal condition of the prediction model. The apparent line represents the theoretical prediction ability, and the bias-corrected line marks the prediction ability of the corrected model. The figure reveals that the above three lines are very close, representing a good prediction.

**Figure 5:**
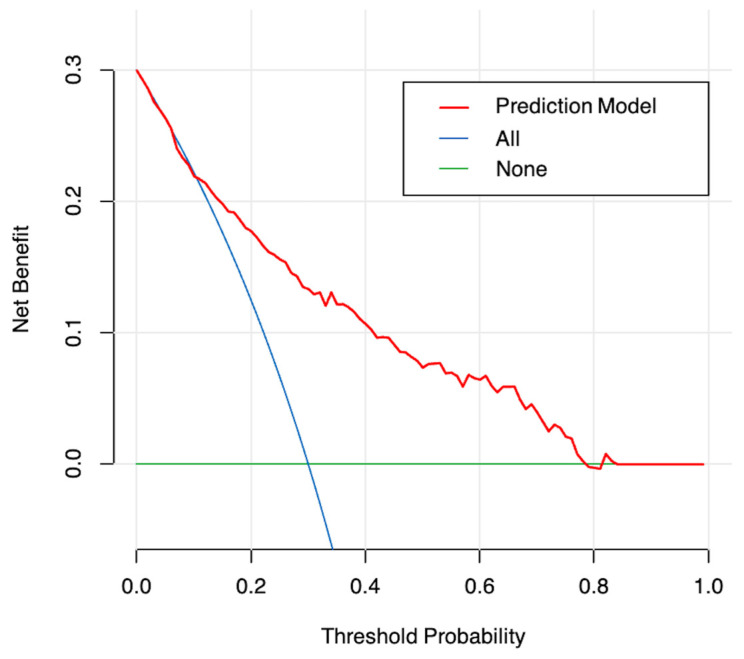
Decision curve analysis for the model. The y-axis measures the net benefit. The red line represents the nomogram. The blue line represents the assumption that all patients undergo FB preoperatively. The green line represents the assumption that no patients undergo FB preoperatively. The decision curve analysis shows that when the probability of threshold is between 11% and 83%, the net benefits of the model for the prediction of positive FB findings are higher compared with other schemes.

An ROC curve was plotted to evaluate the performance of the prediction model (Figure 3). The AUC, sensitivity, and specificity of the model were 0.796 (95% CI, 0.745–0.847), 64.9%, and 82.7%, respectively. The Brier score of the model was 0.156. The AUC and Brier score of the model in internal validation were 0.784 and 0.162, respectively. Calibration curves for the probability of positive FB findings showed convincing concordance between the predicted and actual results, indicating that the model was well-calibrated (Figure 4). Decision curve analysis demonstrated that if the threshold probability of a patient was between 11% and 83%, using the model to predict positive preoperative FB findings added more benefit compared with other schemes (Figure 5).

## 4. Discussion

There is considerable controversy regarding the need to routinely perform preoperative FB in patients with peripheral lung cancer. However, to date, no models have been developed to predict positive FB findings in patients with peripheral lung cancer. Previous studies focusing on the utility of FB made recommendations on the basis of the value of FB in diagnosis and preoperative assessment, but the conclusions were inconsistent since the study populations varied in clinical characteristics [11,13,16,21]. Therefore, we aimed to develop an effective model to predict the probability of positive FB findings, which would help clinicians evaluate the necessity of preoperative FB in patients with peripheral lung cancer.

As an invasive procedure, preoperative FB can assess vocal cord function, examine the status of the bronchi, detect endobronchial tumors, and identify anatomical variations in the bronchi. In addition, FB can obtain histological samples for pathological analysis and examine the bacteriological situation of bronchial secretions [21]. For patients with positive preoperative FB findings, corresponding intervention measures should be taken, including (1) adjusting the surgical plan when an accidental intratracheal tumor is found; (2) using a mucolytic agent to improve ventilation when there is mucosal inflammation or secretion retention in the airway [4,5]; and (3) sampling and culturing the secretions to identify possible pathogenic bacteria and then selecting appropriate antibiotics for treatment [6]. These findings indicate the important role of FB in preoperative diagnosis, airway preparation, and surgical assessment. However, there are also some marked shortcomings of preoperative FB, including discomfort and complications, such as nausea, laryngospasm, bronchospasm, epistaxis, transient hoarseness, fever, cough, mild airway bleeding, severe airway hemorrhage, pneumothorax, severe hypercapnia or hypoxia, arrhythmias, seizures, and cardiac arrest [22,23,24]. Additionally, empirically, many patients who underwent this examination had a negative FB result that did not affect the planned treatment scheme. Therefore, it is necessary to select the patients who are most likely to benefit from FB.

The current study constructed a model to predict the probability of positive FB findings and suggest high-risk patients who are eligible for preoperative FB. The model revealed that male patients with respiratory symptoms, decreased BMI, solid lesions, larger lesions, and lesions located in lower-order bronchi were more likely to have positive FB findings. There are differences between China and Western countries in the clinical practice of FB examination: FB is performed routinely in the endoscopic room before the day of the surgery in China, while it is performed in the process of anesthesia or after anesthesia before the surgery in the US and Europe. According to the model in this study, for low-risk patients in Western countries, FB examination could be simplified in the operation room, which could save time in the operation. In addition, for low-risk patients in China, forgoing this examination could be considered, which could reduce the latency time for the surgery and save an average medical expense of about USD 138 per person.

Gao et al. [25] reported that morphological manifestations and histological types of lung cancer differ between genders: squamous cell carcinoma had a higher incidence in male patients and tended to have a proliferative presentation. Because the location of squamous cell carcinoma in the airway is closer to the central airway [26], it is more likely to be detected. The conclusions of these studies were congruent with the findings of the present study: male patients, who account for a larger proportion of squamous cell carcinoma, had a higher probability of positive preoperative FB tests. Using gender as an independent predictor in the model also indicated that male patients had a higher odds ratio (OR) for positive FB findings compared with female patients.

Ioanas et al. [27] reported that BMI, as a clinical factor, affected bronchial status and found that increased BMI (>25) was an independent risk factor for bronchial colonization, which may lead to positive morphology findings. That finding was explained by decreased diaphragmatic motility in obese patients, which facilitated the accumulation of bronchial secretions and subsequent microbial growth. By contrast, the current study found that decreased BMI was an independent predictor of a positive FB finding. One explanation for this finding may be that patients with decreased BMI could better tolerate FB, and thus more detailed examinations were obtained. Further research is needed to confirm the correlation between BMI and positive FB findings in patients with peripheral lung cancer.

Tsuboi et al. [28] found a significant relationship between tumor size and the number of bronchi involved. Larger tumors are more likely to have bronchial involvement, which increases the likelihood that the bronchoscope reaches the periphery of the tumor, resulting in higher diagnostic sensitivity of FB. Because of our findings and those of previous studies, the predictive model included lesion size as an independent predictor.

A previous study concluded that FB examination was unnecessary in the preoperative assessment of peripheral clinical T1N0 subsolid lung cancer [11]. However, the present study analyzed the relationship between the diagnostic value of FB and lesion type in patients with peripheral lung cancer and found that solid type was a risk factor for positive FB findings. Thus, the lesion type was regarded as a predictor of the model, and a satisfactory predictive efficiency was acquired. Moreover, in view of the observation that lesions located in lower-order bronchi were more likely to have positive FB findings, lesion location in bronchi, including fifth-order bronchi and sixth-order or higher bronchi, were recruited into predictors of the model.

The study does have some limitations. First, the study has the inherent bias of retrospective analysis. Second, only six clinical and radiological factors were involved as predictors of the model, leading to the acquisition of a robust AUC value of 0.796. Finally, owing to sample size limitations, the model was constructed using all samples and then validated using resampling, rather than split-sample development and validation. This was because the former process has higher utilization of samples and is more suited to small sample sizes. Future plans involve the execution of prospective studies that will collect more samples and predictors to improve the performance of the model.

## 5. Conclusions

This study developed a parsimonious clinical prediction model for calculating the probability of positive FB findings in patients with peripheral lung cancers. Males and patients with lower BMI, the presence of respiratory symptoms, larger lesions, solid lesions, and lesions located in lower-order bronchi were associated with increased positive FB findings. The model was validated and proven to have good discrimination. The model has the potential to assist physicians in making clinical decisions about preoperative FB.

## Figures and Tables

**Table 1:** Baseline characteristics of patients with positive and negative FB findings.

Characteristic	Total (*n* = 380)	Positive Findings (*n* = 114)	Negative Findings (*n* = 266)	*p* Value
Gender				<0.001
Male	232 (61.1)	86 (75.4)	146 (54.9)	
Female	148 (38.9)	28 (24.6)	120 (45.1)	
Age in years				0.046
Mean (SD)	61.8 (8.6)	63.2 (8.1)	61.3 (8.7)	
BMI				0.002
Mean (SD)	24.29 (3.47)	23.44 (3.41)	24.65 (3.43)	
Smoking				0.011
Yes	164 (43.2)	61 (53.5)	103 (38.7)	
No	216 (56.8)	53 (46.5)	163 (61.3)	
History of chronic lung diseases				0.246
Yes	84 (22.1)	30 (26.3)	54 (20.3)	
No	296 (77.9)	84 (73.7)	212 (79.7)	
Respiratory symptoms				<0.001
Yes	105 (27.6)	52 (45.6)	53 (19.9)	
No	275 (72.4)	62 (54.4)	213 (80.1)	
Lesion size				<0.001
≤3.0 cm	250 (65.8)	46 (40.4)	204 (76.7)	
>3.0 cm	130 (34.2)	68 (59.6)	62 (23.3)	
Lesion type				<0.001
Solid	303 (79.7)	104 (91.2)	199 (74.8)	
Subsolid	77 (20.3)	10 (8.8)	67 (25.2)	
Lesion location in bronchi				<0.001
Fifth order	234 (61.6)	94 (82.5)	140 (52.6)	
Sixth-order or higher	146 (38.4)	20 (17.5)	126 (47.4)	
Lesion location in lobe				0.867
RUL	118 (31.1)	37 (32.5)	81 (30.5)	
RML	18 (4.7)	7 (6.1)	11 (4.1)	
RLL	90 (23.7)	26 (22.8)	64 (24.1)	
LUL	103 (27.1)	28 (24.6)	75 (28.2)	
LLL	51 (13.4)	16 (14.0)	35 (13.2)	

Abbreviations: SD, standard deviation; RUL, right upper lobe; RML, right middle lobe; RLL, right lower lobe; LUL, left upper lobe; LLL, left lower lobe.

**Table 2:** Univariate logistic regression analysis of prognostic factors in patients with peripheral lung cancer.

Variable	Univariable Analysis	
	Odds ratio (95% Confidence Interval)	*p*-Value
Gender		
Male	Ref.	
Female	0.396 (0.240–0.640)	<0.001
Age	1.027 (1.001–1.055)	0.047
BMI	0.897 (0.835–0.960)	0.002
Smoking		
No	Ref.	
Yes	1.821 (1.171–2.844)	0.008
History of chronic lung diseases		
No	Ref.	
Yes	1.402 (0.833–2.331)	0.196
Respiratory symptoms		
No	Ref.	
Yes	3.371 (2.098–5.442)	<0.001
Lesion size		
≤3.0 cm	Ref.	
>3.0 cm	4.864 (3.055–7.830)	<0.001
Lesion type		
Solid	Ref.	
Subsolid	0.286 (0.133–0.555)	<0.001
Lesion location in bronchi		
Fifth order	Ref.	
Sixth-order or higher	0.236 (0.135–0.398)	<0.001
Lesion location in lobe		
RUL	Ref.	
RML	1.393 (0.479–3.830)	0.526
RLL	0.889 (0.485–1.615)	0.701
LUL	0.817 (0.454–1.461)	0.497
LLL	1.000 (0.485–2.013)	0.998

Abbreviations: Ref., reference; RUL, right upper lobe; RML, right middle lobe; RLL, right lower lobe; LUL, left upper lobe; LLL, left lower lobe.

**Table 3:** Variables identified by logistic multivariable regression analysis.

Variable	Multivariable Analysis		Factors Selected for Model	
	OR (95% CI)	*p*-Value	OR (95% CI)	*p*-Value
Gender				
Male	Ref.		Ref.	
Female	0.454 (0.218–0.944)	0.034	0.582 (0.332–1.006)	0.055
Age	1.019 (0.989–1.051)	0.226		
BMI	0.921 (0.852–0.994)	0.037	0.924 (0.854–0.996)	0.043
Smoking				
No	Ref.			
Yes	0.680 (0.340–1.353)	0.271		
Respiratory symptoms				
No	Ref.		Ref.	
Yes	2.00 (1.140–3.479)	0.015	1.975 (1.129–3.436)	0.016
Lesion size				
≤3.0 cm	Ref.		Ref.	
>3.0 cm	3.126 (1.813–5.427)	<0.001	3.212 (1.869–5.560)	<0.001
Lesion type				
Solid	Ref.		Ref.	
Subsolid	0.486 (0.211–1.040)	0.074	0.505 (0.221–1.070)	0.087
Lesion location in bronchi				
Fifth order	Ref.		Ref.	
Sixth-order or higher	0.234 (0.126–0.418)	<0.001	0.248 (0.135–0.438)	<0.001

Abbreviations: Ref., reference; OR, odds ratio; CI, confidence interval.

## Data Availability

The data that support the findings of this study are available from the corresponding author upon reasonable request.
